# Effects of a cognitive rehabilitation program based on mnemonic skills and memory compensatory strategies for older adults: A pilot study

**DOI:** 10.1097/MD.0000000000029581

**Published:** 2022-08-05

**Authors:** Hyerim Kim, Jimin Lee, Sung Man Chang, Byung-Soo Kim

**Affiliations:** a Department of Psychiatry, Kyungpook National University Hospital, Daegu, South Korea; b Department of Psychiatry, School of Medicine, Kyungpook National University, Daegu, South Korea; c Department of Psychiatry, Kyungpook National University Chilgok Hospital, Daegu, South Korea.

**Keywords:** association learning, cognitive aging, cognitive therapy, mild cognitive impairment, rehabilitation

## Abstract

**Background:**

With the aging of the population, the number of people with age-related memory complaints has also increased. The purpose of this study was to develop a cognitive rehabilitation program based on mnemonic skills and memory compensatory strategies (CRM) and to investigate the effects of CRM in community-dwelling older adults without dementia.

**Methods:**

This study was an open-label, single-arm, pilot study. We developed a CRM program comprising 8 weekly sessions. The study participants consisted of older adults with normal cognitive function and mild cognitive impairment (MCI). They were recruited from eight dementia counseling centers and one senior welfare center. To assess the effects of CRM, we administered the following tests at baseline and after completion of the program: Subjective Memory Complaints Questionnaire, the Short form of Geriatric Depression Scale, the Euro Quality of life–5 Dimension, and the Consortium to Establish a Registry for Alzheimer’s Disease Neuropsychological Assessment Battery.

**Results:**

Thirty-two participants completed the study. Among older adults with normal cognitive function, CRM showed significant improvement in verbal memory function. Among the older adults with MCI, CRM showed significant improvements in language ability, verbal recognition memory, nonverbal memory, attention, and processing speed.

**Conclusion:**

CRM improved cognitive function in two distinct populations, older adults with normal cognitive function and older adults with MCI. Additionally, our preliminary findings suggest that older adults with MCI show cognitive improvement in both the trained and non-trained cognitive domains.

## 1. Introduction

With an increase in the proportion of older adults worldwide, there has been increased attention to the mental health of older adults. Cognitive decline is one of the most common challenges faced by the aging population. As a result, even healthy older adults who do not suffer from dementia often experience memory problems in their everyday lives. Older adults with cognitive complaints are more likely to progress to mild cognitive impairment (MCI) and Alzheimer’s disease (AD) compared with those without cognitive complaints.^[1]^ MCI is considered an intermediate stage between age-related cognitive decline and dementia.^[2]^

However, based on the current knowledge, therapies aimed at restoring cognitive impairments have not yet been successful. Although numerous studies have been conducted to evaluate the efficacy of treatments for dementia, pharmacological interventions have achieved limited success in alleviating symptoms and slowing disease progression.^[3]^ Therefore, it is important to provide evidence for effective non-pharmacological interventions. In particular, cognitive intervention, a non-pharmacological therapy, has the potential to aid in the prevention and treatment of AD and improve cognitive function in older adults who are healthy and those with MCI.^[4,5]^

Cognitive interventions can be classified as cognitive stimulation, cognitive training, or cognitive rehabilitation, depending on the methods that are employed.^[6]^ Cognitive training involves the structured practice of standardized tasks that are developed to improve specific cognitive functions, such as working memory, attention, or executive function.^[7]^ Cognitive stimulation refers to participation in non-specific activities that stimulate cognitive and social functioning. These activities include orientation reality, reminiscence therapy, paper folding, creating a topiary or ceramic item, and playing a musical instrument.^[8]^ It has been reported that cognitive stimulation and cognitive training improve cognitive function in healthy older adults and older adults with MCI.^[9]^ However, as their therapeutic value is restricted to the trained cognitive areas, these interventions are limited in their effectiveness for reducing the discomfort of daily life and improving the quality of independent living. Cognitive rehabilitation is defined as the rehabilitation of people with cognitive impairments and it focuses on not only improving or maintaining cognitive function related to the performance of everyday tasks but also compensating for impairments and supporting independent living.^[6]^

Cognitive rehabilitation was originally developed primarily through the training of young people with traumatic brain injury but it has also been applied to people with cognitive impairments such as MCI or dementia.^[6]^ Cognitive rehabilitation has been used for years in patients with traumatic brain injury or stroke, and several evidence-based studies have demonstrated its effectiveness.^[10]^ Although several studies have shown that cognitive rehabilitation is effective in people with cognitive impairments,^[11,12]^ there are relatively few well-designed studies on the development and effectiveness of programs that involve cognitive rehabilitation in people with MCI. Cognitive rehabilitation is considered as one of the most effective interventions in slowing down the progression of cognitive decline,^[13]^ and as these interventions have been administered relatively recently, additional studies are needed to assess and verify their therapeutic effects.

Cognitive rehabilitation methods include restorative approaches and compensatory methods.^[14]^ Restorative approaches are based on retained abilities and employ a range of techniques to promote learning and relearning. Compensatory methods are based on a range of aids to support function and overcome the limitations resulting from cognitive impairments.^[15]^ According to a methodological review of cognitive rehabilitation, this approach uses not only learning strategies, such as mnemonic skills, but also compensatory strategies that use external memory aids.^[16]^ Mnemonic skills are systematic procedures for enhancing memory and cognitive tools that facilitate the organization and association of new information.^[16]^ One of the most prominent mnemonic skills is the method of loci, an ancient technique used extensively by Greek and Roman orators.^[17]^ The method of loci utilizes well-established memories of visuospatial routes. During encoding, the to-be-remembered lists are visualized at fixed locations within a route, then they are mentally retraced during recall.^[18]^ Memory recall is enhanced when the method of loci is applied to memorize word lists.^[19,20]^ Cognitive interventions, including mnemonic skills, facilitate learning and memory enhancement in healthy older adults and people with MCI.^[21,22]^ These skills also improve quality of life by promoting active transfer to everyday cognitive tasks.^[23]^ Compensatory strategies use environmental cues to enhance memory and everyday functioning using external memory aids, such as notes, calendars, and cell phones.^[24]^ People with MCI can improve their functional ability and memory self-efficacy using a notebook or calendar.^[25]^

Previous studies have shown that cognitive interventions can improve the functioning of trained cognitive domains. However, only a few studies have assessed the aspects of daily functioning or quality of life; the results have been inconsistent across studies and the effect sizes were small.^[26,27]^ In addition, cognitive interventions focused on memory compensatory strategies improved daily functioning but did not show a clear improvement in cognitive functions.^[25]^ Therefore, we developed a cognitive rehabilitation program that combined mnemonic skills and memory compensatory strategies to overcome the limitations of previous studies, which have only independently used mnemonic skills or compensatory strategies.^[16,21,22,24,25]^ In particular, we focused on training to memorize a password for a bankbook, a door-lock password, and a person’s name that older adults could easily forget in their daily life using mnemonic skills rather than training tasks related to specific cognitive domains such as memory, attention, and executive function. We also tried to improve daily function by employing memory compensatory strategies using various tools, such as calendars or cell phones, which are widely available in the vicinity. This made it easier for older adults to apply the strategies in daily life. This pilot study aimed to reduce memory problems that can cause discomfort in everyday life and improve the quality of life among community-dwelling older adults. This study also evaluated the effects of our proposed cognitive rehabilitation program on subjective memory discomfort, objective cognitive function, depressive mood, and quality of life.

## 2. Methods

### 2.1. Study participants

The required sample size was calculated using a moderate effect size (*d* = 0.50), based on the effect size reported in a previous study.^[28]^ A minimum of 14 samples was required for each group to achieve 80% power at a 5% significance level. Considering the relatively high age group and a drop-out rate of 20%, we decided to recruit approximately 40 participants for this pilot study.

The study participants were recruited from a senior welfare center and eight community-based dementia counseling centers in Daegu from July 2018 to August 2018. We enrolled individuals over the age of 60 with either normal cognitive function or MCI. MCI was diagnosed according to clinical and neuropsychological information based on Petersen’s criteria.^[29]^ Older adults with MCI had subjective memory complaints and an objective cognitive impairment that was determined when the score of any of the nine neuropsychological tests (except the Trail Making Test B [TMT-B] in the Consortium to Establish a Registry for Alzheimer’s Disease Neuropsychological Assessment Battery [CERAD-K]) was less than −1.5 standard deviation of the age-, gender-, and education-adjusted norms for Korean older adults. We excluded patients diagnosed with dementia using the diagnostic criteria of the Diagnostic and Statistical Manual of Mental Disorders, 5th edition (DSM-5). We also excluded participants with major psychiatric disorders, neurological disorders, or severe physical or speech disturbances that could affect cognitive function or make it difficult to attend the programs. We included participants who were regularly taking medications, such as sedatives or anticholinergics at stable doses, and these participants were instructed to maintain their medications for the duration of the program. This was done to avoid confusion about the effects of CRM that are attributed to changes in medication status. All participants attended the program for free.

### 2.2. Cognitive rehabilitation program

The CRM consisted of eight weekly sessions. Each session lasted approximately 50 minutes and was composed of two parts; the first part focused on mnemonic skills and the second part focused on memory compensatory strategies. The contents of each session are summarized in Table 1. Occupational therapists were trained in a 4-hour educational course for the program, and they subsequently conducted the program in each center. Additionally, both the older adults with normal cognitive function and older adults with MCI trained in the centers together.

**Table 1:** Contents of each cognitive rehabilitation program session based on mnemonic skills and memory compensatory strategies.

Session	Mnemonic skills	Memory compensatory strategies
1	Word association with images 1	Using a cellphone camera
2	Word association with images 2	Managing schedules using a calendar, a note, or a board
3	Method of loci	Using an alarm
4	Method of body parts	Using a timer
5	Memorizing numbers	Taking important things when going out
6	Face-name association 1	Using a storage box
7	Face-name association 2	Taking medications without forgetting
8	Review: various ways of memorizing using both mnemonic skills and memory compensatory strategies together	

The mnemonic skills sessions included word association with images, the method of loci, the method of body parts, numeric conversion, and face–name association. The occupational therapists educated the participants about mnemonic skills using detailed examples and allowed them to rehearse immediately. The participants repeatedly learned methods of association (word association with images, the method of loci, and the method of body parts), which are the basis of mnemonic skills, and these were practiced over several sessions. Using the method of association, the participants were able to remember a list of items (e.g., a grocery list). The first few sessions were intended for practicing and mastering the method of association sufficiently. In the succeeding sessions, participants learned and practiced how to memorize items, such as a password for a bank book, a door-lock password, and family birthdays by converting numbers to images. Participants also learned and practiced how to memorize a person’s name using face-name association, which links the salient features of appearance and name by applying the method of association. In the final session, participants practiced how to improve their memory and recall in various situations by applying the mnemonic skills that they had previously learned from the program. The learning process was repeated so that the participants would be able to learn and apply mnemonic skills to everyday life.

In the second part of the session, participants learned memory compensatory strategies using memory aids such as notebooks, calendars, cell phones, timers, storage boxes, and pillboxes. In older people with memory decline, external aids can be more effective than memory alone. The participants were taught methods about how to manage their schedules using calendars, remember unfamiliar places (e.g., parking lots) or important persons by taking pictures with cell phones, remember important items by placing them in specific, consistent locations (e.g., a basket or drawer), and remember to take medications by using pillboxes. At the end of the session, the participants were assigned some homework, which is reviewed in the following session. The participants were encouraged to practice these skills repeatedly in their daily lives.

### 2.3. Neuropsychological assessments

All participants were evaluated by clinical psychologists or trained nurses before and immediately after the program.

The CERAD-K,^[30]^ which consists of 10 neuropsychological tests that assess five cognitive domains (attention, memory, language, visuospatial, and executive function), was used to evaluate objective cognitive function. The CERAD-K was developed by translating the English version of the CERAD^[31]^ clinical and neuropsychological assessment batteries into Korean. This takes into consideration the cultural and linguistic differences between English and Korean users. The CERAD-K is as reliable and valid as the English version of the CERAD. The MMSE^[32]^ in the CEARD neuropsychological battery was translated except for items related to reading and writing since a large number of older adults in Korea are illiterate. These items were replaced with two items based on the Korean version of the MMSE (MMSE-K).^[33]^ Cronbach’s alpha value for the Mini-Mental Status Examination in the Korean version of the CERAD Assessment Packet (MMSE-KC) was 0.92. The 15-item Boston Naming Test (BNT) was constructed by extracting 5 items for each lexical frequency (high, medium, and low) based on 60 items of the Korean version-BNT (K-BNT) standardized in Korea.^[34]^ Language function was assessed by the Categorical Fluency Test (CFT) and the 15-item BNT; general cognitive function was assessed by the MMSE-KC; verbal memory was assessed by the Word List Memory Test (WLMT), the Word List Recall Test (WLRT), and the Word List Recognition Test (WLRcT); nonverbal memory was assessed by the Constructional Recall Test (CRT); visuospatial function was assessed by the Constructional Praxis Test (CPT); attention and processing speed were assessed by the Trail Making Test A (TMT-A); executive function was assessed by TMT-B.

Subjective memory complaints (SMC) were evaluated using the Subjective Memory Complaints Questionnaire (SMCQ), which has been validated and adapted for the Korean population.^[35]^ The Cronbach’s α coefficient and intra-class correlation (ICC) coefficients of the SMCQ were 0.864 and 0.828, respectively. The SMCQ is a self-rating scale that comprises 15 items with “yes” or “no” answers that include SMC about general and everyday memory. A higher score indicates increased severity of SMC.

Depressive mood was evaluated by administering the Korean version of the Short form of Geriatric Depression Scale (SGDS-K), which was standardized by Cho et al.^[36]^ The SGDS-K, which consists of 15 items, is designed to assess mood during the week preceding the test as a score of 0 or 1, with a range of outcome scores from 0 to 15. The higher the score of the SGDS-K, the greater the severity of depression; the cut-off point for depressive symptoms is 8.^[36]^ The Cronbach’s α coefficient of the SGDS-K was 0.886.

We also administered the Euro Quality of life-5 Dimension (EQ-5D) questionnaire to measure health-related quality of life. The EQ-5D is a standardized measure of health status that was developed by the Euro Quality of Life group and consists of a descriptive system and the EQ Visual Analog Scale (EQ-VAS).^[37]^ The EQ-5D descriptive system comprises 5 dimensions: mobility, self-care, usual activities, pain/discomfort, and anxiety/depression. Each dimension is rated at three levels; no problems, some problems, and extreme problems using a coded 3-point scale ranging from 1 to 3. The EQ-VAS is a vertical form visual analog scale ranging from 0 for the lowest health status to 100 for the best health status. The EQ-5D is a simple and useful test for the elderly in terms of time and cognition. In this study, we used the Korean version of the EQ-5D that was developed and validated for the Korean population.^[38,39]^ The ICCs of the EQ-5D and EQ-VAS were 0.751 and 0.767, respectively, and the kappa values ranged from 0.455 to 0.772. The EQ-5D index was calculated by applying the valuation weights used in the National Health and Nutrition Survey. ^[40]^

### 2.4. Statistical analysis

This was a small, open-label, preliminary study and therefore, parametric statistical analyses were not suitable. Participants who did not meet the inclusion criteria and those who dropped out were excluded from the study. Only those who completed the program were subsequently analyzed. Baseline characteristics were compared using the Mann–Whitney *U* test for continuous variables and Fisher’s exact test for categorical variables. We analyzed the effects of CRM in older adults with normal cognitive function and older adults with MCI. The Wilcoxon signed-rank test was used to evaluate the effects of CRM on SMC, objective cognitive function, depressive mood, and quality of life by assessing the differences between the scores before and after CRM training.

In addition, to analyze the effects of CRM, the effect size was calculated using the Z value of the Wilcoxon signed-rank test. The formula used was as follows^[41]^:


$$\begin{array}{l} r\;=\;\frac{Z}{\sqrt{n}} \end{array}$$

Cohen’s guidelines for rare that a large effect is.5, a medium;

effect is.3, and a small effect is.1 (Coolican, 2009, p. 395);

Cohen’s guidelines for rare that a large effect is.5, a medium;

effect is.3, and a small effect is.1 (Coolican, 2009, p. 395);

Cohen’s guidelines for *r* state that a large effect is 0.5, medium effect is 0.3, and small effect is 0.1.^[41,42]^

All statistical analyses were performed using Statistical Package for the Social Sciences version 21.0 software (SPSS IBM, Armonk, NY, USA). For all analyses, *P* values <.05 were considered statistically significant.

### 2.5. Ethical considerations

The study protocol was reviewed and approved by the institutional review board of Kyungpook National University (approval No. 2018-0081). Written informed consent was obtained from each participant at the time of enrollment.

## 3. Results

### 3.1. Sample characteristics

**Figure 1.**
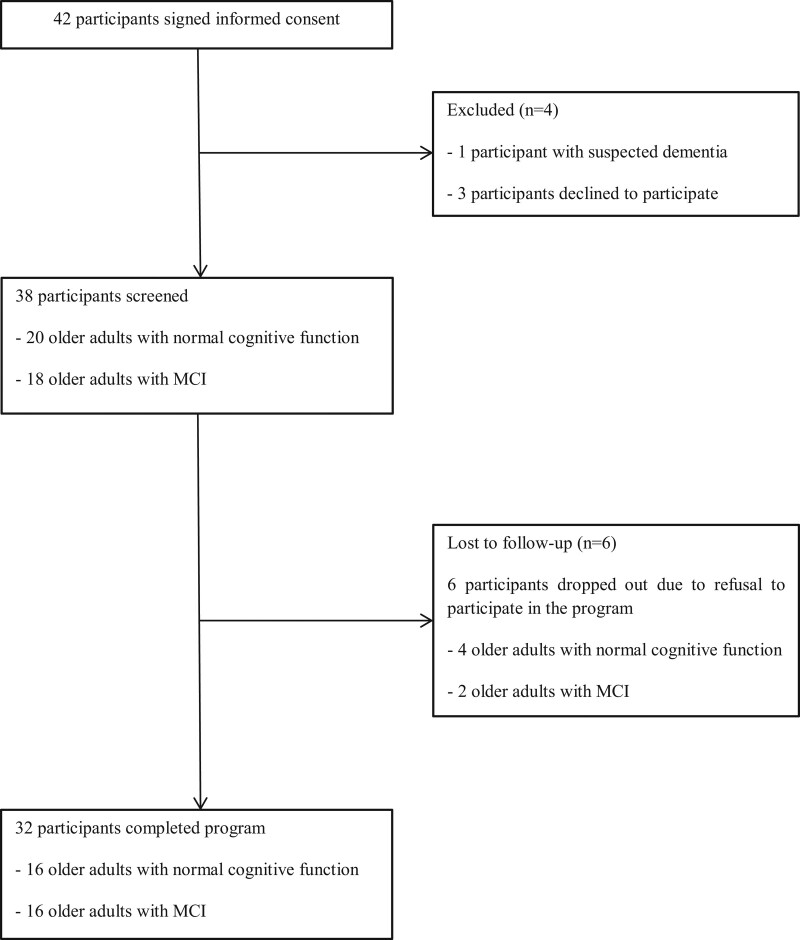
Flowchart of study participants. We enrolled community-dwelling older adults aged over 60 years with normal cognitive function or with MCI. A total of 42 participants were recruited; among them, one participant with suspected dementia was excluded and three participants declined to participate before the program started. Thirty-eight participants (20 with normal cognitive function and 18 with MCI) participated in the program and six participants (four with normal cognitive function and two with MCI) dropped out because of refusal to participate in the program. Thirty-two participants finally completed the program and were assessed after the program.

A total of 42 participants were recruited; among them, one participant with suspected dementia was excluded and three participants declined to participate before the program started. A total of 38 participants (20 with normal cognitive function and 18 with MCI) participated in the program and 6 participants (4 with normal cognitive function and 2 with MCI) dropped out because of their refusal to participate in the program (Fig. 1). Therefore, a total of 32 participants (16 with normal cognitive function and 16 with MCI) completed the study. The baseline demographic and clinical characteristics of the participants are summarized in Table 2. There were no significant differences between older adults with normal cognitive function and older adults with MCI except for the CPT scores.

**Table 2:** Baseline demographic and clinical characteristics.

Characteristics	CRM (n = 32)		
	Normal (n = 16)	MCI (n = 16)	*P*
Age (y)	68.63 ± 4.40	70.88 ± 5.34	.445
Female	13 (81.3%)	13 (81.3%)	1.000
Education (y)	8.38 ± 4.29	9.50 ± 4.02	.642
SMCQ	4.94 ± 3.44	6.56 ± 3.12	.171
SGDS-K	4.31 ± 5.17	1.94 ± 2.44	.323
EQ-5D	0.82 ± 0.13	0.90 ± 0.06	.056
EQ-VAS	71.13 ± 16.30	67.63 ± 22.15	.926
CERAD-K			
CFT	13.00 ± 2.66	12.81 ± 3.73	.956
BNT	11.63 ± 2.60	10.75 ± 2.46	.305
MMSE-KC	27.88 ± 2.22	27.63 ± 2.45	.752
WLMT	20.06 ± 3.11	20.06 ± 3.45	1.000
WLRT	6.56 ± 1.41	6.13 ± 1.71	.724
WLRcT	9.19 ± 1.28	8.69 ± 1.66	.381
CPT	10.38 ± 1.15	6.50 ± 3.83	.002*
CRT	7.13 ± 3.05	7.94 ± 3.44	.402
TMT-A (s)	66.00 ± 49.92	100.63 ± 86.00	.073

### 3.2. Effects of CRM on outcome measures in older adults with normal cognitive function

Intra-group comparisons of the older adults with normal cognitive function are shown in Table 3. When pre- and post-test scores were compared, CRM showed significant improvements in the scores of WLMT (*P* = .016) and WLRT (*P* = .049). In terms of the effect size of CRM, the effect sizes for WLMT and WLRT were 0.426 and 0.348, respectively, both indicating medium effects.

**Table 3:** Comparisons between pre and post outcomes in older adults with normal cognitive function* (n = 16).

Outcome measure	Pre	Post	Z	*P*	*r*
SMCQ	4.94 ± 3.44	4.69 ± 3.59	−0.398	.691	−0.070
SGDS-K	4.31 ± 5.17	3.50 ± 5.30	−1.178	.239	−0.208
EQ-5D	0.82 ± 0.13	0.85 ± 0.13	−1.140	.254	−0.202
EQ-VAS	71.13 ± 16.30	74.69 ± 17.84	−1.029	.304	−0.182
CERAD-K					
CFT	13.00 ± 2.66	14.13 ± 4.10	−1.000	.317	−0.177
BNT	11.63 ± 2.60	11.94 ± 2.24	−0.855	.393	−0.151
MMSE-KC	27.88 ± 2.22	27.31 ± 1.92	−1.920	.055	−0.339
WLMT	20.06 ± 3.11	22.44 ± 3.31	−2.407	.016†	−0.426
WLRT	6.56 ± 1.41	7.31 ± 1.82	−1.968	.049†	−0.348
WLRcT	9.19 ± 1.28	9.69 ± 0.79	−1.841	.066	−0.325
CPT	10.38 ± 1.15	10.56 ± 0.81	−0.351	.726	−0.062
CRT	7.13 ± 3.05	7.44 ± 3.65	−0.616	.538	−0.109
TMT-A (s)	66.00 ± 49.92	60.94 ± 30.08	−0.284	.776	−0.050

### 3.3. Effects of CRM on outcome measures in older adults with MCI

Intra-group comparisons of the older adults with MCI are presented in Table 4. Comparison of pre- and post-test scores within-group revealed significant improvements in BNT (*P* = .009), WLRcT (*P* = .016), CPT (*P* = .005), CRT (*P* = .012), and TMT-A (*P* = .033). In terms of the effect size of CRM, the effect sizes of BNT, WLRcT, CPT, CRT, and TMT-A were 0.464, 0.424, 0.496, 0.442, and 0.377, respectively, all indicating medium effects.

**Table 4:** Comparisons between pre and post outcomes for each program group in the elderly with mild cognitive impairment* (n = 16).

Outcome measure	Pre	Post	*Z*	*P*	*r*
SMCQ	6.56 ± 3.12	5.69 ± 3.44	−1.188	.235	−0.210
SGDS-K	1.94 ± 2.44	1.31 ± 1.62	−0.787	.431	−0.139
EQ-5D	0.90 ± 0.06	0.88 ± 0.09	−1.024	.306	−0.181
EQ-VAS	67.63 ± 22.15	70.94 ± 10.99	−0.710	.478	−0.126
CERAD-K					
CFT	12.81 ± 3.73	13.81 ± 2.71	−1.191	.234	−0.211
BNT	10.75 ± 2.46	12.06 ± 2.72	−2.623	.009†	−0.464
MMSE-KC	27.63 ± 2.45	27.94 ± 1.65	−0.668	.504	−0.118
WLMT	20.06 ± 3.45	20.63 ± 3.65	−1.031	.303	−0.182
WLRT	6.13 ± 1.71	7.00 ± 1.41	−1.767	.077	−0.312
WLRcT	8.69 ± 1.66	9.56 ± 0.81	−2.401	.016‡	−0.424
CPT	6.50 ± 3.83	9.13 ± 2.68	−2.807	.005‡	−0.496
CRT	7.94 ± 3.44	9.38 ± 2.75	−2.501	.012‡	−0.442
TMT-A (s)	100.63 ± 86.00	73.38 ± 36.72	−2.131	.033‡	−0.377

## 4. Discussion

This study assessed the impact of CRM on subjective cognitive discomfort, objective cognitive function, quality of life, and depressive mood in older adults. CRM improved verbal memory in older adults with normal cognitive function and various cognitive functions (language ability, verbal recognition memory, nonverbal memory, attention, and processing speed) in older adults with MCI.

Regarding cognitive function, there were significant improvements in WLMT and WLRT scores for older adults with normal cognitive function and the BNT, WLRcT, CPT, CRT, and TMT-A scores for older adults with MCI. The effect size of CRM was medium in both groups. Previous studies have shown that mnemonic skills are linked to improvements in memory functions, such as recall and recognition memory.^[21,43,44]^ CRM not only demonstrated an improvement in memory function but also in language ability, attention, and processing speed. The transfer of training is the generalization of the training effect from one trained domain to another nontrained domain(s). The transfer is an important factor in determining the effectiveness of cognitive interventions and their potential to reduce or delay the incidence of dementia.^[45]^ Transfer of training occurs at multiple levels including transfer to non-trained tasks within the same cognitive domain (near transfer) or other cognitive domains (far transfer), and generalization of effects on everyday functioning.^[5]^ The mnemonic skills of CRM primarily focus on training verbal episodic memory. In older adults with normal cognitive function, only near transfer occurred. In older adults with MCI, there occurred far transfer where the effects of CRM could be transferred to untrained cognitive domains (such as language ability, attention, and processing speed) other than memory as well as near transfer. These results are consistent with previous studies, suggesting that training mnemonic skills is effective for older adults with MCI and can improve different cognitive domains.^[16,26,27]^ Mnemonic skills training in CRM, such as the method of loci, requires the participant’s attention and visuospatial ability. Through this form of training, various cognitive domains are stimulated, which improves episodic memory, as well as other cognitive domains. In addition, memory compensatory strategies would further strengthen memory function in daily life by allowing the participants to learn internal strategies using external aids. The effect size of CRM was medium in both groups, which is consistent with the results of previous meta-analyses where the effect size of cognitive intervention was found to range from small to moderate.^[26,28]^ These findings suggest that CRM could be an effective intervention for both older adults with normal cognitive function and older adults with MCI and that various cognitive domain functions might improve, especially among older adults with MCI.

Concerning general cognitive ability and general function, CRM did not significantly improve MMSE-KC, SMCQ, SGDS-K, EQ-5D, or EQ-VAS scores in older adults with normal cognitive function or MCI. This finding indicates that the efficacy of CRM cannot be transferred to everyday functioning. Contrary to our expectations, the SMCQ scores were not significantly better. As learning mnemonic skills is both time-consuming and requires effort, some participants may have experienced difficulty and frustration while mastering these new skills; these could undermine their subjective competence and result in negative ratings in SMCQ scores. In previous studies, cognitive interventions including mnemonic skills or memory compensatory strategies have shown near and far transfer.^[9]^ However, the results of previous studies were inconsistent in terms of the generalization of effects on improving daily functioning, such as quality of life, subjective cognitive function, depressive mood, and activities of daily living.^[12,46]^ Although our findings demonstrated improvements in cognitive function, there were no significant improvements in SMC or quality of life. This might be due to the small sample size, short training duration, lack of long-term follow-up, or importantly, the low level of functional impairment in older adults with MCI. Mnemonic skills and memory compensatory strategies were found to improve memory function. Memory compensatory strategies are easier to learn and apply than mnemonic skills, which might help older adults with MCI enhance their memory function and self-efficacy. Applying these strategies to everyday tasks through repetitive CRM training could relieve some of the issues in daily life caused by memory decline. Subsequently, this would improve the quality of life. Therefore, it is expected that the generalization of the effects of CRM will be demonstrated through further research with larger sample populations.

This study had several limitations that should be acknowledged. First, since this was a pilot study, the number of participants was relatively small. Therefore, the results should be interpreted with caution because the statistical power of the tests was reduced and the likelihood of producing a type II error was higher. Second, this study was conducted with no control group, which makes the interpretation of the effects difficult. Although CRM improved cognitive function in this study, further studies with the control group are needed to confirm this effect. Third, no follow-up assessments were performed. The long-term effect of CRM on cognitive function could be important in delaying the onset of dementia because older adults with MCI have a high probability of developing dementia. Fourth, we did not assess health behaviors such as smoking and alcohol consumption, which could affect the performance of cognitive interventions.^[47]^ In addition, physical conditions, such as hypertension and diabetes, which are risk factors for cognitive impairment,^[48]^ were not considered. However, in most previous studies, these physical conditions were not considered moderators of the effect of cognitive interventions, and a systematic review and meta-analysis found that demographic variables such as age, education, and cognitive status did not significantly impact intervention efficacy.^[26]^ This pilot study evaluated the short-term effect of CRM; therefore, it was assumed that these factors would not have a significant effect on the program effect.

In conclusion, our results demonstrated that CRM improved cognitive function in both trained and untrained cognitive domains, although it did not improve daily functioning. These results are promising, but the small sample size limits the generalization of our findings. Nevertheless, rather than training for a task in a specific cognitive domain, it is important to highlight that we attempted to make it easier for older adults to apply strategies in real life by training mnemonic skills and memory compensatory strategies that are focused on memory problems that are frequently experienced in the daily life. Therefore, additional randomized, controlled studies with larger sample sizes are needed to confirm the effects of CRM and its maintenance as a non-pharmacological intervention strategy for older adults with cognitive impairments. These results are expected to contribute to the development of increasingly effective cognitive rehabilitation programs for older adults with cognitive impairment.

## Author contributions

Conceptualization: Hyerim Kim, Sung Man Chang, Byung-Soo Kim. Data curation: Hyerim Kim, Jimin Lee, Byung-Soo Kim. Formal analysis: Hyerim Kim, Byung-Soo Kim. Investigation: Hyerim Kim, Byung-Soo Kim. Methodology: Hyerim Kim, Jimin Lee, Byung-Soo Kim. Supervision: Sung Man Chang, Byung-Soo Kim. Validation: Sung Man Chang, Byung-Soo Kim

Writing – original draft: Hyerim Kim. Writing – review & editing: Hyerim Kim, Byung-Soo Kim.

## Footnotes

* Significant at *P* < .01.

* By Wilcoxon signed-rank test.

† Significant at *P* < .05.

* By Wilcoxon signed-rank test.

† Significant at *P* < .05.

‡ Significant at *P* < .01.

## References

[1] Jessen F Wiese B Bachmann C. Prediction of dementia by subjective memory impairment: effects of severity and temporal association with cognitive impairment. Arch Gen Psychiatry. 2010;67:414–22. 20368517 10.1001/archgenpsychiatry.2010.30

[2] Albert MS DeKosky ST Dickson D. The diagnosis of mild cognitive impairment due to Alzheimer’s disease: recommendations from the National Institute on Aging and Alzheimer’s Association workgroup. Alzheimer’s Dement. 2011;7:270–9. 21514249 10.1016/j.jalz.2011.03.008 PMC3312027

[3] Soldan A Pettigrew C Cai Q. Cognitive reserve and long-term change in cognition in aging and preclinical Alzheimer’s disease. Neurobiol Aging. 2017;60:164–72. 28968586 10.1016/j.neurobiolaging.2017.09.002 PMC5679465

[4] Chalfont G Milligan C Simpson J. A mixed methods systematic review of multimodal non-pharmacological interventions to improve cognition for people with dementia. Dementia (London). 2020;19:1086–130. 30193536 10.1177/1471301218795289 PMC7180318

[5] Kelly ME Loughrey D Lawlor BA. The impact of cognitive training and mental stimulation on cognitive and everyday functioning of healthy older adults: a systematic review and meta-analysis. Ageing Res Rev. 2014;15:28–43. 24607830 10.1016/j.arr.2014.02.004

[6] Bahar-Fuchs A Clare L Woods B. Cognitive training and cognitive rehabilitation for mild to moderate Alzheimer’s disease and vascular dementia. Cochrane Database Syst Rev. 2013(6):CD003260. 23740535 10.1002/14651858.CD003260.pub2 PMC7144738

[7] Bahar-Fuchs A Martyr A Goh AM. Cognitive training for people with mild to moderate dementia. Cochrane Database Syst Rev. 2019;3:CD013069. 30909318 10.1002/14651858.CD013069.pub2 PMC6433473

[8] Woods B Aguirre E Spector AE Orrell M. Cognitive stimulation to improve cognitive functioning in people with dementia. Cochrane Database Syst Rev. 2012(2):CD005562. 10.1002/14651858.CD005562.pub2 22336813

[9] Mewborn CM Lindbergh CA. Cognitive interventions for cognitively healthy, mildly impaired, and mixed samples of older adults: a systematic review and meta-analysis of randomized-controlled trials. Neuropsychol Rev. 2017;27:403–39. 28726168 10.1007/s11065-017-9350-8

[10] Cicerone KD Goldin Y Ganci K. Evidence-based cognitive rehabilitation: systematic review of the literature from 2009 through 2014. Arch Phys Med Rehabil. 2019;100:1515–33. 30926291 10.1016/j.apmr.2019.02.011

[11] Regan B Wells Y Farrow M. MAXCOG-maximizing cognition: a randomized controlled trial of the efficacy of goal-oriented cognitive rehabilitation for people with mild cognitive impairment and early alzheimer disease. Am J Geriatr Psychiatry. 2017;25:258–69. 28034509 10.1016/j.jagp.2016.11.008

[12] Clare L Kudlicka A Oyebode JR. Individual goal-oriented cognitive rehabilitation to improve everyday functioning for people with early-stage dementia: a multicentre randomised controlled trial (the GREAT trial). Int J Geriatr Psychiatry. 2019;34:709–21. 30724405 10.1002/gps.5076 PMC6593854

[13] Amieva H Robert PH Grandoulier AS. Group and individual cognitive therapies in Alzheimer’s disease: the ETNA3 randomized trial. Int Psychogeriatr. 2016;28:707–17. 26572551 10.1017/S1041610215001830

[14] Clare L Bayer A Burns A. Goal-oriented cognitive rehabilitation in early-stage dementia: study protocol for a multi-centre single-blind randomised controlled trial (GREAT). Trials 2013;14:152–152. 23710796 10.1186/1745-6215-14-152 PMC3680175

[15] Huckans M Hutson L Twamley E. Efficacy of cognitive rehabilitation therapies for mild cognitive impairment (MCI) in older adults: working toward a theoretical model and evidence-based interventions. Neuropsychol Rev. 2013;23:63–80. 23471631 10.1007/s11065-013-9230-9 PMC3640648

[16] Hampstead BM Gillis MM Stringer AY. Cognitive rehabilitation of memory for mild cognitive impairment: a methodological review and model for future research. J Int Neuropsychol Soc. 2014;20:135–51. 24331156 10.1017/S1355617713001306

[17] Bower GH. Analysis of a mnemonic device. Am Sci. 1970;58:496–510.

[18] Dresler M Shirer WR Konrad BN. Mnemonic training reshapes brain networks to support superior memory. Neuron 2017;93:1227–1235.e6. 28279356 10.1016/j.neuron.2017.02.003 PMC5439266

[19] Brooks JO 3rd Friedman L Pearman AM. Mnemonic training in older adults: effects of age, length of training, and type of cognitive pretraining. Int Psychogeriatr. 1999;11:75–84. 10189601 10.1017/s1041610299005608

[20] Yesavage JA Sheikh JI Friedman L. Learning mnemonics: roles of aging and subtle cognitive impairment. Psychol Aging 1990;5:133–7. 2317292 10.1037//0882-7974.5.1.133

[21] Hampstead BM Sathian K Moore AB. Explicit memory training leads to improved memory for face-name pairs in patients with mild cognitive impairment: results of a pilot investigation. J Int Neuropsychol Soc. 2008;14:883–9. 18764984 10.1017/S1355617708081009

[22] Hampstead BM Sathian K Phillips PA. Mnemonic strategy training improves memory for object location associations in both healthy elderly and patients with amnestic mild cognitive impairment: a randomized, single-blind study. Neuropsychology 2012;26:385–99. 22409311 10.1037/a0027545 PMC3348454

[23] Bottiroli S Cavallini E Dunlosky J. Self-guided strategy-adaption training for older adults: transfer effects to everyday tasks. Arch Gerontol Geriatr. 2017;72:91–8. 28609674 10.1016/j.archger.2017.05.015

[24] Andrewes DG Kinsella G Murphy M. Using a memory handbook to improve everyday memory in community-dwelling older adults with memory complaints. Exp Aging Res. 1996;22:305–22. 8872083 10.1080/03610739608254013

[25] Greenaway MC Duncan NL Smith GE. The memory support system for mild cognitive impairment: randomized trial of a cognitive rehabilitation intervention. Int J Geriatr Psychiatry. 2013;28:402–9. 22678947 10.1002/gps.3838 PMC3766962

[26] Mewborn CM Lindbergh CA. Cognitive interventions for cognitively healthy, mildly impaired, and mixed samples of older adults: a systematic review and meta-analysis of randomized-controlled trials. Neuropsychol Rev. 2017;27:403–39. 28726168 10.1007/s11065-017-9350-8

[27] Chandler M Parks A Marsiske M. Everyday impact of cognitive interventions in mild cognitive impairment: a systematic review and meta-analysis. Neuropsychol Rev. 2016;26:225–51. 27632385 10.1007/s11065-016-9330-4 PMC5048589

[28] Sherman DS Mauser J Nuno M. The Efficacy of Cognitive Intervention in Mild Cognitive Impairment (MCI): a meta-analysis of outcomes on neuropsychological measures. Neuropsychol Rev. 2017;27:440–84. 29282641 10.1007/s11065-017-9363-3 PMC5754430

[29] Petersen RC Roberts RO Knopman DS. Mild cognitive impairment: ten years later. Arch Neurol. 2009;66:1447–55. 20008648 10.1001/archneurol.2009.266 PMC3081688

[30] Lee JH Lee KU Lee DY. Development of the Korean version of the Consortium to Establish a Registry for Alzheimer’s Disease Assessment Packet (CERAD-K): clinical and neuropsychological assessment batteries. J Gerontol B Psychol Sci Soc Sci. 2002;57:P47–53. 11773223 10.1093/geronb/57.1.p47

[31] Morris JC Heyman A Mohs RC. The Consortium To Establish A Registry For Alzheimer’s Disease (CERAD): I. Clinical and neuropsychological assessment of Alzheimer’s disease. Neurology 1989;39:1159–65. 2771064 10.1212/wnl.39.9.1159

[32] Folstein MF Folstein SE McHugh PR. “Mini-mental state”: a practical method for grading the cognitive state of patients for the clinician. J Psychiatr Res. 1975;12:189–98. 1202204 10.1016/0022-3956(75)90026-6

[33] Park JH Park YN Ko HJ. Modification of the mini-mental state examination for use with the elderly in a non-western society. Part II: cutoff points and their diagnostic validities. Int J Geriatr Psychiatry. 1991;6:875–82.

[34] Kim H Na D. Korean Version-Boston Naming Test. Seoul: Hakjisa. 1997.

[35] Youn JC Kim KW Lee DY. Development of the subjective memory complaints questionnaire. Dement Geriatr Cogn Disord. 2009;27:310–7. 19252402 10.1159/000205512

[36] Cho MJ Bae JN Suh GH. Validation of geriatric depression scale, Korean version (GDS) in the assessment of DSM-III-R major depression. J Korean Neuropsychiatr Assoc. 1999;38:48–63.

[37] EuroQol G. EuroQol--a new facility for the measurement of health-related quality of life. Health Policy 1990;16:199–208. 10109801 10.1016/0168-8510(90)90421-9

[38] Kim SH Jo MW Lee JW. Validity and reliability of EQ-5D-3L for breast cancer patients in Korea. Health Qual Life Outcomes. 2015;13:203. 26694964 10.1186/s12955-015-0399-x PMC4689040

[39] Kim MH Cho YS Uhm WS. Cross-cultural adaptation and validation of the Korean version of the EQ-5D in patients with rheumatic diseases. Qual Life Res. 2005;14:1401–6. 16047514 10.1007/s11136-004-5681-z

[40] Lee YK Nam HS Chuang LH. South Korean time trade-off values for EQ-5D health states: modeling with observed values for 101 health states. Value Health 2009;12:1187–93. 19659703 10.1111/j.1524-4733.2009.00579.x

[41] Fritz CO Morris PE Richler JJ. Effect size estimates: current use, calculations, and interpretation. J Exp Psychol Gen. 2012;141:2. 21823805 10.1037/a0024338

[42] Cohen J. Statistical Power Analysis For The Behavioral Sciences. Routledge; 2013.

[43] Cavallini E Pagnin A Vecchi T. Aging and everyday memory: the beneficial effect of memory training. Arch Gerontol Geriatr. 2003;37:241–57. 14511850 10.1016/s0167-4943(03)00063-3

[44] Bier N Van Der Linden M Gagnon L. Face-name association learning in early Alzheimer’s disease: a comparison of learning methods and their underlying mechanisms. Neuropsychol Rehabil. 2008;18:343–71. 18569747 10.1080/09602010701694723

[45] Sala G Aksayli ND Tatlidil KS. Near and far transfer in cognitive training: A second-order meta-analysis. Collabra: Psychology 2019;5.

[46] Reijnders J van Heugten C van Boxtel M. Cognitive interventions in healthy older adults and people with mild cognitive impairment: a systematic review. Ageing Res Rev. 2013;12:263–75. 22841936 10.1016/j.arr.2012.07.003

[47] Silva GM Almeida NL Souto JJ. Santos NAd. Does chronic smoking affect performance on a go/no-go task? Curr Psychol. 2021:1–9.

[48] Rodrigue KM Rieck JR Kennedy KM. Risk factors for beta-amyloid deposition in healthy aging: vascular and genetic effects. JAMA Neurology 2013;70:600–6. 23553344 10.1001/jamaneurol.2013.1342 PMC3968915

